# Wiring Up the Newest Part of the Cortex

**DOI:** 10.1371/journal.pbio.1001355

**Published:** 2012-06-26

**Authors:** Charles Q. Choi

**Affiliations:** Freelance Science Writer, New York, New York, United States of America

**Figure:**
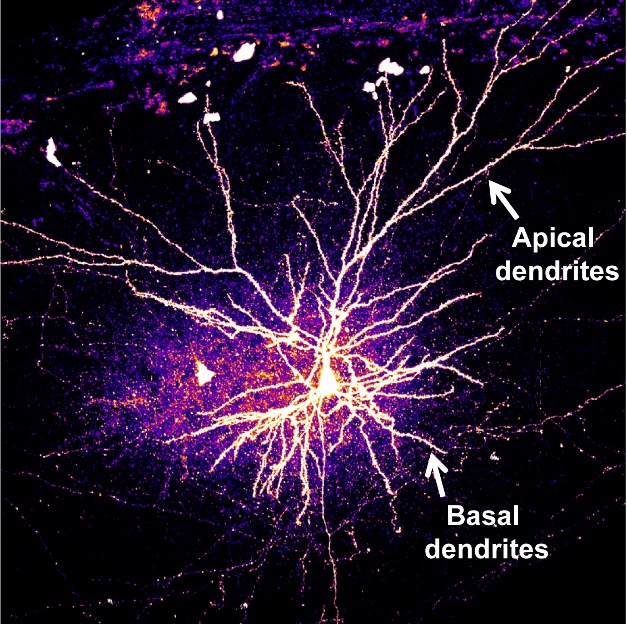
Apical and basal dendrites of cortical pyramidal neurons exhibit distinct shapes, and basal dendrites retract when Ras-Epac2 signaling is disrupted.

The most common type of cell in the neocortex, the center of higher mental function in humans, is the pyramidal neuron. The dendrites of these neurons are the structures that receive signals from other neurons, and they form specific shapes called arbors. Disruptions in the shape and complexity of these dendritic arbors are seen in disorders such as schizophrenia and autism. There are two types of dendrites defined by location: apical dendrites, which stretch far from the cell bodies toward the surface of the brain, and basal dendrites found close to the cell bodies. These regions appear distinct in function, but how the arbors of basal versus apical dendrites are maintained is unclear. In this issue of *PLoS Biology*, Peter Penzes and his colleagues have discovered a molecular pathway involved specifically in regulating basal dendrites.

The scientists focused on a protein called Epac2, which helps regulate Rap, a Ras-like small GTPase highly enriched in the adult brain and dendrites. By knocking down Epac2 in a specific population of pyramidal neurons in the mouse cortex, they found that this led to a significant reduction in both the number and length of basal dendritic branches.

Interestingly, four rare amino acid coding variants have been identified in the EPAC2 gene in people with autism, suggesting they could help yield insights on the protein's function. Therefore, the authors overexpressed one of these mutant proteins in cultured rat cortical neurons, and again found reduced basal dendritic complexity and length. This single amino-acid mutation, seen in four people with autism from two families, is located within the Ras association domain of Epac2. The small GTPase Ras has been implicated in neuronal morphogenesis, so this new result suggested that Epac2 may be regulating basal dendritic arbors via its interaction with Ras. The authors tested this by first showing that Epac2 normally interacted with Ras in cultured rat cortical neurons, but that the mutant Epac2 had significantly impaired interactions with Ras. Importantly, disrupting Ras pharmacologically in cultured neurons also reduced basal dendritic complexity and length.

Penzes and his colleagues conclude that Epac2 enables crosstalk between the Ras and Rap signaling pathways to maintain basal dendrite complexity. When they looked at the expression of these proteins in different regions of pyramidal neurons, they found that each protein was less concentrated in basal dendrites than apical ones, which suggests that these lower levels of Epac2, Ras, and Rap could make basal dendrites more vulnerable to impairments such as mutations. Furthermore, these low levels might also reflect the fact that basal dendrites are built to be more variable and dynamic than apical dendrites, due to their distinct functions in signal integration and information processing.

The investigators say future work will involve analyzing the function of Epac2 in vivo, at both the circuit and behavioral level. They add that further studies should also assess the role of Epac2 in human disorders—more rare Epac2 mutations may be found in people with autism, further elucidating its function. It may be that Epac2 is part of a signaling network, as Penzes and his colleagues have previously shown that Epac2 interacts with neuroligins, important autism susceptibility molecules.

These findings show how disease-linked mutations identified in clinical research can, in a “reverse translational” strategy, can yield insights into basic research matters such as neuronal connectivity and morphology.

**Srivastava DP, Woolfrey KM, Jones KA, Anderson CT, Smith KR, et al. (2012) An Autism-Associated Variant of Epac2 Reveals a Role for Ras/Epac2 Signaling in Controlling Basal Dendrite Maintenance in Mice. doi:10.1371/journal.pbio.1001350**

